# BPEX Pig Health Scheme: a useful monitoring system for respiratory disease control in pig farms?

**DOI:** 10.1186/1746-6148-7-82

**Published:** 2011-12-30

**Authors:** Hannah R Holt, Pablo Alarcon, Martina Velasova, Dirk U Pfeiffer, Barbara Wieland

**Affiliations:** 1 Veterinary Epidemiology & Public Health Group, Department of Veterinary Clinical Sciences, Royal Veterinary College, London, UK

## Abstract

**Background:**

Respiratory diseases account for significant economic losses to the UK pig industry. Lesions indicative of respiratory disease in pig lungs at slaughter e.g. pneumonia and pleuritis are frequently recorded to assess herd health or provide data for epidemiological studies. The BPEX Pig Health Scheme (BPHS) is a monitoring system, which informs producers of gross lesions in their pigs' carcasses at slaughter, enabling farm-level decisions to be made. The aim of the study was to assess whether information provided by the BPHS regarding respiratory lesions was associated with respiratory pathogens in the farm, farm management practices and each other.

**Results:**

BPHS reports were obtained from a subset of 70 pig farms involved in a cross-sectional study conducted in 2008-09 investigating the epidemiology of post-weaning multi-systemic wasting syndrome. The reports were combined with data regarding the presence/absence of several pathogens in the herd and potential farm-level risk factors for respiratory disease. Principal component analysis (PCA) performed on BPHS reports generated three principal components, explaining 71% of the total variance. Enzootic pneumonia score, severe pleurisy and acute pleuropneumonia had the highest loadings for the principal component which explained the largest percentage of the total variance (35%) (BPHS component 1), it was thought that this component identifies farms with acute disease. Using the factor loadings a score for each farm for BPHS component 1 was obtained. As farms' score for BPHS component 1 increased, average carcass weight at slaughter decreased. In addition, farms positive for H1N2 and porcine reproductive and respiratory disease virus (PRRSV) were more likely to have higher levels of severe and mild pleurisy reported by the BPHS, respectively.

**Conclusions:**

The study found statistical associations between levels of pleurisy recorded by BPHS at slaughter and the presence H1N2 and PRRSV in the herd. There is also some evidence that farms which submit pigs with these lesions may have reduced productivity. However, more research is needed to fully validate the scheme.

## Background

Respiratory diseases are a major problem in intensive pig farming [1] accounting for significant economic losses due to increased mortality, morbidity and treatment costs and reduced growth rates, feed conversion efficiency and carcass quality [2,3]. Enzootic pneumonia and pleuritis (or pleurisy) are common respiratory diseases in the UK pig industry, lesions indicative of which are frequently found in pig lungs at slaughter [4-6].

Slaughter data has been used in epidemiological studies [4,7-9] and several infectious and non-infectious factors have been associated with lung lesions at slaughter. Presence of particular pathogens such as *Actinobacillus pleuropneumoniae *(APP) and *Mycoplasma hyopneumoniae *(*M. hyopneumoniae*) and management factors including increasing herd size, increased stocking density and being a farrow-to-finish herd have been associated with increased risk of lung lesions [8,10,11], whilst implementing an all-in-all-out system has been demonstrated to be protective [12].

Routine inspection of pig lungs at slaughter is also used to monitor herd health. The BPEX Pig Health Scheme (BPHS), run by BPEX, a division of the Agriculture and Horticulture Development Board (AHDB), is a monitoring system in England and Wales which aids producers and veterinarians in farm-level decision making. It is a voluntary scheme launched in July 2005, predominantly funded by a levy collected from pig producers, pharmaceutical company sponsors and the Department for Environment, Food and Rural Affairs (DEFRA) (until 2008). The BPHS takes place in participating abattoirs on certain days. On assessment days producers receive a report of every batch of pigs they send to the abattoir detailing the farm-level frequency of gross pathological lesions observed in their pigs at slaughter.

The lungs are removed from each carcass as part of the 'pluck' and hung in a line parallel to the carcass; BPHS assessors perform a mild palpation of the lungs and move them around in order to assess the whole surface of the organ. An external contractor is responsible for the recruitment and management of the BPHS assessors; in total fifty-five trained veterinary assessors have participated in the BPHS at some point [13]. Although, this could lead to observer bias, BPHS attempts to minimise this by having at least two assessors per abattoir and each assessor working in at least two abattoirs [13]. In addition annual standardisation days are conducted and on occasions, regional coordinates carry out simultaneous scoring with assessors to identify potential misclassifications [13].

Table 1 summarises the lesions of interest in the current study, which are scored as part of the BPHS. The descriptions and typical causes are those the BPHS provides as part of its guidelines to farmers and veterinarians. In addition to respiratory lesions and pericarditis the BPHS records peritonitis, abscesses, pyaemia, tail bites, papular dermatitis, milk spots and hepatic scarring, however, these were not of interest in the current study.

**Table 1:** Summary of the BPHS scores of a batch level report and their typical causes, according to BPEX guidelines [13,14]

Score	Description	Typical Causes
Enzootic pneumonia (EP) like lesion score	EP-like lesions in the anterior lobes of the lungs. The report shows the average score (minimum = zero: no lesions, maximum = 55), for all lungs examined. Each pair of lungs is divided into 7 lobes; the cranial lobes, cardiac lobes, diaphragmatic lobes and a single accessory (intermediate) lobe. Depending on the level of disease, each cranial and cardiac lobe is scored from 0 to 10 and the cranial areas of the diaphragmatic lobes and intermediate lobe are scored from 0 to 5.	*M. hyopneumoniae *
Viral-like pneumonia	Lesions of viral pneumonia: lobular pattern with consolidation, rubbery texture, congestion or collapsed areas. Percentage of pigs and the number of individuals with viral-like pneumonia is given.	Porcine reproductive and respiratory virus (PRRSV), swine influenza (SI), porcine circovirus 2 (PCV2)
Chronic pleuropneumonia	Bronchopneumonia with overlying pleurisy, usually affecting caudal or middle lung lobes. A severe form of pleurisy usually associated with APP. The percentage of pigs and the number of individuals with chronic pleuropneumonia-like lesions. The presence of old lesions suggests past infection and variable immunity in herds with endemic pleuropneumonia.	APP
Acute pleuropneumonia	As above but fresh, active lesions. The report presents the percentage of pigs and the actual number of individuals with acute pleuropneumonia-like lesions. The presence of new, active lesions indicates recent infection.	APP
Mild or localised pleurisy	The percentage and number of pigs with any discrete area of parietal or visceral pleurisy. The affected area of the lung may be stuck to the chest wall. The presence of pleurisy provides evidence that a lung infection had occurred prior to slaughter.	*Haemophilus parasuis Mycoplasma *spp., *Actinobacillus *spp., *Streptococcus *spp. *Bordetella bronchiseptica*, etc.
Severe or extensive pleurisy	The percentage and number of pigs with pleurisy involving in excess of approximately 20% of the total lung area, is classed as extensive.	as above, but more likely to be APP
Pericarditis	The number and percentage of pigs with pericarditis (inflammation of the pericardium).	*Haemophilus parasuis *and other causes of pleurisy

Although producers consider the BPHS useful for farm-level decision making, the BPHS has never been formally validated [15]. In addition, as BPHS assessors are not the official meat inspectors the lungs cannot be incised and the assessment is visual only. The aim of the current study was to investigate whether there was any evidence that the information provided by BPHS regarding lung lesions in pigs at slaughter reflects the respiratory disease situation on the farm. Associations between BPHS lung lesions present at slaughter and common respiratory pathogens in the farm, farm management practices, productivity and the relationships between the different BPHS lesions were assessed.

## Methods

### Study population and data collection

Data used in the current study were obtained from a previous cross-sectional study investigating PMWS and the methods have been described in detail elsewhere [16,17]. Convenience sampling was used in the study; farms enrolled in a PCV2 vaccination scheme were contacted and asked if they would be willing to take part in the study. Further farms were recruited through veterinary practitioners. In total 147 farms were involved in that study and during farm visits, a detailed questionnaire was administered collecting information regarding farm management practices, environment, health problems on the farm, medication, vaccination protocols, reproduction management and production parameters. In addition, blood samples from twenty pigs; six weaners, six growers (11 to 14 weeks old), six finishers (> 14 weeks old) and two sows were collected. Sample size for this study was calculated in order to detect at least one infected animal with 95% confidence, assuming a 10% prevalence of an infection and close contact between age groups. As part of the questionnaire, farmers were asked if they were enrolled in the BPHS and if so, permission to obtain their BPHS data and use it for further study was obtained.

BPHS quarterly reports were obtained for the quarter in which the farm was sampled or, if this was not available, the quarter closest to the date of the farm visit. If there were no data available within two quarters of the farm visit then the farm was excluded from the study. Pigs are assessed by BPHS on specified days at participating abattoirs. On assessment days, every other pig submitted for slaughter from farms enrolled in the scheme is assessed, until a maximum of 50 pigs per batch are sampled. Each quarterly report summarises the results of all pigs assessed from that farm over a three month period by providing the number, and percentage, of pigs with each lesion, or in the case of EP, the average EP score of all pigs assessed during that quarter (Table 1). According to Stärk (2000) there is a consensus that at least 30 pig lungs need to be submitted to reliably estimate the prevalence of pneumonia in pig lungs at slaughter [18], therefore if any farms submitted less than 30 pigs for slaughter during that quarter, they were also excluded from the study.

Of the 147 farms which took part in the PMWS study, 70 farms were enrolled in the BPHS scheme, had BPHS reports available within six months of the date of blood sampling, gave permission for their data to be used in the current study and had a complete dataset. All farms were farrow-to-finish herds, although in some farms production took place on multiple sites. Twenty-five farms had more than one site but only one finishing site (where growers and finishers were kept). Therefore all samples and BPHS reports came from the same sites. Six farms had 2-6 finishing units, in these farms samples were taken from the largest finishing unit; these units were in close proximity and management practices were similar in all sites.

### Serological Testing

Blood samples were tested for *M. hyopneumoniae*, APP, porcine parvovirus (PPV), porcine reproductive and respiratory syndrome virus (PRRSV), porcine circovirus 2 (PCV2) and swine influenza (SI). Laboratory testing was carried out as described in previous studies (Table 2). However, weaners' and sows' serology and PCR results were excluded from the analysis as maternal antibodies may be present in weaners and sows may have antibodies from infection a long time ago and are not sent to slaughter.

**Table 2:** Respiratory pathogens tested for and tests used [1,16,17,19-22]

Pathogen	Test type	Test manufacturer	Information
APP	ELISA for serotypes 3,6 & 8	Swinecheck^® ^(Biovet, Saint-Hyacinthe, Quebec, Canada)	Sensitivity: 90 to 99% Specificity: 95 to 99%
PCV2	PCR	Protocol by Yang et al. 2007	Detection threshold: 10^3^-10^11 ^million copies DNA per reaction
PRRSV (unvaccinated farms)	ELISA	BioBest Elisa (Biobest laboratories, United Kingdom)	Sensitivity: > 95% and Specificity: > 95%
PRRSV (vaccinated farms)	RT-PCR	AcuPig^® ^PRRSV (AnDiaTec GmbH & Co., Germany)	Detection threshold: 100 to 200 viral particles in 1 ml blood
SI	Haemagglutination inhibition (HI) test	Veterinary Laboratories Agency, United Kingdom (OIE reference laboratory)	Office International des Epizooties (OIE) standard

Many farms vaccinated piglets against *M. hyopneumoniae *(70%) and Porcine Parvovirus (85.7%). Due to the low numbers of unvaccinated farms, the *M. hyopneumoniae *and PPV results were excluded from the statistical analysis, however, whether farms vaccinated against *M. hyopneumoniae *and PPV or not were included as explanatory variables. No pigs in the study were vaccinated against SI or APP. The ELISA for APP detects serotypes 3, 6 and 8 which are the predominant serotype in the UK [19], accounting for approximately 88% infections (personal communication, Paul Langford, Imperial College London).

Approximately half (52.9%) of farms vaccinated their piglets against PRRSV and the ELISA test for PRRSV cannot distinguish between natural antibodies and antibodies produced by the vaccine. Therefore, samples from pigs which were vaccinated against PRRSV were re-tested using RT-PCR. Unvaccinated farms were classified as positive if at least one grower or finisher tested positive for PRRS using the ELISA test, and vaccinated farms were classified as positive if at least one grower or finisher tested positive using the RT-PCR. In addition, because farms in the study had problems with PMWS, PCV2 antibodies were widespread throughout herds therefore PCV2 was tested for using PCR in order to have enough farms with a low number of positive pigs for statistical analysis.

### Data management and principal component analysis

Data from the cross-sectional PMWS study were stored in Microsoft Access 2007; relevant data were extracted and combined with BPHS data in Microsoft Excel 2007. Descriptive statistics were obtained and data were exported to STATA v.9 for further statistical analysis. All analyses were performed at farm level.

Principal component analysis (PCA) was performed with all the BPHS variables. The aim of the PCA was to identify patterns in the dataset, assess potential collinearity and investigate the possibility of condensing the variables into fewer principal components, which could be used as variables in statistical analyses. Components which had an eigenvalue > 1 (which is proportional to the amount of variance accounted for by that component) were retained and any variables which had a loading value < 0.4 in all components were removed. Finally, if the retained principal components explained at least 70% of the variance they were considered suitable. A score for the component which explained the largest percentage of the variance was then calculated for each farm in proportion to the loading factors for each variable. These scores were then transformed in order to use them in the statistical analysis. Firstly they were made positive by adding the minimum value plus one; the minimum score for BPHS component 1 was -1.733 therefore 2.74 was added to all the BPHS component 1 score, these scores were then logged.

### Risk factor analysis

Risk factor analysis was performed at the farm level and associations with EP score, severe pleurisy and mild pleurisy from farms' BPHS reports were assessed. As the prevalence of mild and severe pleurisy and average EP score varied substantially between farms, these variables were grouped into three categories. A previous BPEX case-control classified farms as controls if the percentage of pigs from the farm with pleurisy at slaughter was five or less and classified farms as a case if more than ten percent had pleurisy [6]. Therefore for severe and mild pleurisy farms with 5% or less pigs affected were classified as not having a problem with mild/severe pleurisy, farms that submitted > 5% to ≤ 10% of pigs affected with mild/severe pleurisy were classified as having a moderate problem and farms that submitted > 10% of pigs with mild/severe pleurisy were considered to have a severe problem.

BPHS guidelines classify pigs as having mild EP if their average score is between one and five. Therefore farms were classified as not having a problem with EP when their average EP score was less than one and as having a mild EP problem if their average EP score ranged from one to 4.99. Pigs with an EP score between five and ten are classed as having moderate EP therefore farms with an average EP score of five or more were classified as having a moderate EP problem (there were no farms with an average score of ten or more).

In addition, associations with a continuous outcome, average carcass weight (obtained from the PMWS study) were also investigated; this measure was used as a proxy for productivity. Pigs are sent to slaughter systematically, either at a precise age or weight, and average age was included as a fixed effect in the model to control for its confounding effects. Statistical associations with acute pleuropneumonia, chronic pleuropnemonia and viral pneumonia were not assessed as very few farms submitted pigs with these lesions and most farms that did only submitted one or two pigs.

Associations between the outcomes and other BPHS variables, respiratory pathogens (Table 2) and farm management practices were assessed. Although only EP score, severe pleurisy and mild pleurisy were used as outcomes in statistical analysis, all other lesions scored by BPHS (including non-respiratory lesions) were used as exposures in the models. Farm management practices investigated included herd size, type of ventilation, stocking density, implementation of an all-in-all out system, separation of age groups, bringing gilts onto the farm, etc. Univariable analysis was initially performed between each outcome and each explanatory variable. Linear regression was performed for average carcass weight and ordinal logistic regression for the BPHS outcome variables which had three ordered categories.

All variables associated with the outcomes with a *p-*value of 0.2 or less in the univariable analysis were included in a multivariable-linear model (average carcass weight) or multivariable ordinal logistic regression model (BPHS variables) in order to investigate the association with the outcomes, controlling for the possible confounding effects of other variables. The model was optimised using a backwards step-wise procedure, variables were removed one by one, starting with the highest *p*-value, and a likelihood ratio test performed; variables were permanently excluded when the likelihood ratio test gave a *p*-value of > 0.05.

## Results

### Summary statistics

The number of pigs examined by BPHS within farms in the current study ranged from 30 to 281 per quarter, with a median of 50 pigs. The median value for average EP score of pig farms in the current study was 1.14; 41.4% of farms had an average EP score of one to 4.99 and 14.3% of farms had an average EP score of five or more. Severe pleurisy was the most common BPHS lesion recorded at slaughter with a prevalence of severe pleurisy of 5.01 to 10% in 25.7% of farms and of more than 10% in 27.1% of farms. Furthermore, 18.6% of farms had mild pleurisy in 5.01 to 10% of pigs assessed and 11.4% of farms had mild pleurisy in more than 10% of pigs assessed.

With respect to pleuropneumonia, 12.9% and 8.6% of farms had acute and chronic pleuropneumonia in at least one pig assessed, respectively. In addition, 12.9% of farms submitted pigs with viral-type lesions. In the case of pericarditis, 25.7% of farms had a within-farm prevalence of pericarditis ranging from 5.01 to 10% and 8.6% of farms had a within-farm prevalence ≥ 10%.

From the results of the blood tests, 39 (55.7%) farms were seropositive for APP, 22 (31.4%) were (sero)positive for PRRSV and 39 (55.7%) farms were seropositive for at least one strain of swine influenza (SI). Nineteen (27.1%) farms were seropositive for H1N1 and 35 (50%) farms were seropositive for H1N2. Only 24.3% of farms had < 25% of their pigs test positive for PCV2, 31.7%, 22.9% and 15.7% of farms had 25 to 49.9%, 50 to 74.5% and > 75% of their pigs test positive for PCV2, respectively.

### Results of the principal component analysis

Pearson's correlation coefficients between variables are presented in Table 3. The results indicate that severe pleurisy was moderately correlated with EP (0.47) and acute pleuropneumonia (0.40); however, many of the variables exhibit some independence.

**Table 3:** Correlation between prevalence of different types of lesions recorded by BPHS at slaughter

	EP score	Viral	Acute PP	Chronic PP	Mild pleurisy	Severe pleurisy	Pericar-ditis
**EP score**	1.00						
**Viral**	0.32	1.00					
**Acute PP**	0.27	0.14	1.00				
**Chronic PP**	0.14	0.11	0.35	1.00			
**Mild pleurisy**	-0.02	0.18	-0.05	-0.004	1.00		
**Severe pleurisy**	0.47	0.16	0.40	0.08	0.15	1.00	
**Pericarditis**	0.26	-0.01	0.13	-0.20	0.21	0.35	1.00

Using principal component analysis; three components accounting for 71% of the total variance exhibited by the respiratory lesions scored by BPHS at slaughter were identified and retained (Table 4). These three components explained 35% (BPHS component 1), 19% (BPHS component 2) and 17% (BPHS component 3) of the total variance. EP score, severe pleurisy and acute pleuropneumonia had the highest factor loadings for BPHS component 1, with moderate factor loadings for viral-type lesions and pericarditis. Farms with high scores for BPHS component 2 submitted a high proportion of pigs with mild pleurisy, but without acute pleuropneumonia and farms with high scores for BPHS component 3 had viral type lesions without pericarditis.

**Table 4:** Characteristics of retained components from PCA of BPHS variables

Components (Eigenvalues)	BPHS 1 (2.75)	BPHS 2 (1.32)	BPHS 3 (1.12)	Unexplained Variance
EP score	**0.51**	-0.20	0.13	38.5%
Severe pleurisy	**0.56**	-0.04	-0.17	31.7%
Mild pleurisy	0.17	**0.81**	0.10	18.4%
Acute pleuropneumonia	**0.40**	**-0.42**	-0.07	45.2%
Viral-type lesions	0.31	0.12	**0.79**	13.4%
Pericarditis	0.37	0.33	**-0.56**	25.7%

### Risk factor analysis for lesions recorded by BPHS

Several relationships between respiratory lesions recorded by BPHS and non-infectious and infectious factors were identified (Table 5). The test for the assumption of proportional odds gave *p*-values of 0.26, 0.57 and 0.89 for the EP, severe pleurisy and mild pleurisy models, respectively. As the null hypothesis was that the assumption of proportional odds was suitable for the data, all ordinal models were considered sound.

**Table 5:** Risk factors for BPHS lesions identified from the multivariable analysis

Variables	OR (95% CI)	Model fit
*Outcome: Average EP score group*		
Severe pleurisy group	3.44 (1.84 to 6.42)	*R^2 ^*= 0.228
*Outcome: Severe pleurisy*		
EP score group	3.67 (1.62 to 8.32)	
Pericarditis Group	2.80 (1.12 to 7.01)	*R^2 ^*= 0.386
H1N2 positive	4.27 (1.48 to 12.3)	
Finish meat pigs	3.87 (1.16 to 12.9)	
*Outcome: Mild Pleurisy*		
PRRS positive	3.91 (1.34 to 11.3)	*R^2 ^*= 0.120

### Associations with BPHS variables

Using ordinal logistic regression, for every unit increase in severe pleurisy group, the odds of farms being in a higher group for average EP score increased by 3.44 (95% confidence interval (CI):1.84 - 6.42). Severe pleurisy was the only variable associated with EP score in farms in the current study, although in the univariate analysis farms which vaccinated against EP had 2.97 (95% CI: 1.07 to 8.25) times the odds of being in a higher group for average EP score. Every unit increase in pericarditis group appeared to increase the odds of farms being in a higher group for severe pleurisy by 2.80 (95% CI: 1.12 to 7.01). Although acute pleuropneumonia was strongly related to severe pleurisy it was excluded from the model because it is a severe form of pleurisy associated with APP. In addition to BPHS variables, farms seropositive for H1N2 and farms that finished their own pigs had increased odds of being in a higher group for severe pleurisy. Farms seropositive for PRRS had increased odds of being in a higher group for mild pleurisy. Whether or not farms vaccinated against PRRS was not associated with the presence of lung lesions.

### Associations with components identified from PCA and productivity

The results of associations with productivity are presented in Table 6; increases in BPHS component 1 and keeping weaners and finishers together were associated with decreases in farms' average carcass weight.

**Table 6:** Results of multivariable analysis for variables associated with BPHS components and productivity (carcass weight)

Variables	**Coef. (95% CI**)	Model fit
BPHS component 1 Weaners and finishers	-4.42 (-6.92 to -1.70) 3.35 (-6.38 to -0.33)	*R^2 ^*= 0.895
Average age at slaughter	0.93 (0.02 to 0.94)	

## Discussion

The BPHS is used by farmers to monitor their within-farm prevalence of respiratory lesions, to identify potential outbreaks of respiratory disease in their herd and to assess whether different management interventions e.g. vaccination are successful at reducing lung lesions present at slaughter. To our knowledge this is the first study to objectively assess the usefulness of BPHS data for the monitoring of respiratory diseases, other than EP. The study found several statistical associations with respiratory lesions examined by BPHS at slaughter.

There are several limitations in the current study; pigs which were blood sampled and tested for respiratory pathogens were not the same pigs examined for gross lesions at slaughter. Lesions may vary substantially between pigs and over time therefore the results must be interpreted with extreme caution. However, the BPHS data used was as close as possible to the time of blood sampling and respiratory pathogens often persist within farms [23,24]. In order to evaluate the BPHS further, testing would need to be carried out on pigs undergoing BPHS examination and the results of these tests linked with their BPHS reports. Despite these issues the study does not attempt to draw causal inferences between lesions present at slaughter and circulating pathogens in the herd; but simply identifies potential associations which could be used as hypotheses in further studies.

Another drawback is that not all respiratory pathogens were tested for, therefore associations between the pathogens present and BPHS lesions may be confounded by the presence of other respiratory pathogens. Also, analysis was performed at the farm-level and farms were simply classified as positive/negative for most pathogens. The presence of gross lesions in lungs at slaughter is likely to be influenced by within-herd prevalence and pathogen load, in addition to presence/absence of infection.

Management practices of the farm often play an important role in respiratory disease; farms that finished their own meat pigs on site and kept weaners and finishers together had increased odds of submitting pigs with severe pleurisy and an increased score for BPHS component 1, respectively. If further studies were carried out investigating which management factors are associated with respiratory lesions present and slaughter, this information could be used to provide farmers with useful recommendations for tackling respiratory disease problems. Many studies focus on the relationship between management factors and pathogens present in the herd. However, if it could be shown that certain interventions can improve carcass quality this could help farmers become more responsive to their BPHS reports.

In the univariate analysis, farms which vaccinated against *M. hyopneumoniae *had higher EP scores. Farmers will use their BPHS reports to aid their decision on whether or not to vaccinate against *M. hyopneumoniae*, with higher scoring farms more likely to start vaccinating. Hence, in the current study, vaccinating and non-vaccinating farms are unlikely to be comparable in terms of initial level of disease and it is not possible to draw strong conclusions on the impact of vaccination. Previous studies have found reduced lung lesions of EP in vaccinated pigs at slaughter [25], however, in a study by Villarreal et al (2011) this reduction was non-significant [26]. In this study it is likely that *M. hyopneumoniae *vaccination has reduced the impact of the pathogen to some extent; however farms still have higher EP scores compared to non-vaccinating farms, which are less likely to have had problems with EP. It is important to remember that *M. hyopneumoniae *vaccination will not eliminate *M. hyopneumoniae *but reduce extent of clinical signs and lesions of EP at slaughter. Further the presence/absence of certain risk factors will play a role in disease.

EP-like lung lesion score is frequently used to assess herd health with regards to *M. hyopneumoniae*, but information regarding other lesions present at slaughter could be made more useful. Although, it is not possible to make an etiological diagnosis on the basis of slaughterhouse examination only and many respiratory problems, pleurisy in particular, are multifactoral [27]. However this study, and others [6], relating gross lung lesions to the presence of certain respiratory pathogens in farms and farm management practices, could be used to give producers and veterinarians some direction when tackling respiratory disease in response to their BPHS reports. The results of the current study and a project conducted by BPEX indicate that PRRS and H1N2 may be associated with the presence of mild and severe pleurisy in pig lungs at slaughter, respectively [6]. In addition, this study highlights the high proportion of farms seropositive for H1N2 (50%). However, with regards to pleurisy; SI and PRRSV do not currently appear in the BPHS guidelines for producers interpreting their reports.

Although, H1N2 was associated with severe pleurisy in the current study, H1N1 was not associated with any of the lung lesions. This was unexpected as H1N1 is the most common isolate identified from passive surveillance in the UK, therefore it was thought that H1N1 may be more pathogenic [20]. The BPHS is carried out in clinically healthy pigs therefore, perhaps if pigs infected with H1N2 are less likely to exhibit clinical signs they may be more likely to reach slaughter. However, the results of the study suggest these pigs may still have lung lesions present at slaughter, which may result in economic losses.

For a typical 10% prevalence of pleurisy at batch level, losses are estimated to be approximately £2.26 per pig due to reduced carcass weight and/or increased time to slaughter [6]. Within farms in the current study, up to 44% of pigs assessed had severe pleurisy, therefore costs to these producers are likely to be high. The study also found that increased scores for BPHS component 1 were associated with a decrease in the average carcass weight indicating that farms with a high prevalence of EP, severe pleurisy and acute pleuropneumonia (plus viral pneumonia and pericarditis) may have an overall reduction in productivity compared with farms that do not. The BPHS report for 2005 to 2008, suggests there has been a linear trend towards reduction in mean monthly prevalence of pleurisy in participating farms [6], suggesting participation in the BPHS scheme may have economic benefits for producers, although these benefits are difficult to quantify.

Although the recording of lesions is abattoir based, so only pigs of slaughter age are included in the sample, there is potential for BPHS data to support disease surveillance in pigs at a national level. BPEX already monitor the prevalence of the lesions scored by BPHS in participating farms, producing reports detailing changes in the prevalence of lesions. A sudden increase in the prevalence of lesions could act as an early warning system for an emerging disease or indicate a new outbreak of an old one. Spatial analysis of the BPHS data could identify severely affected areas and local/regional outbreaks of disease, providing numbers of farms assessed by the scheme in each area was controlled for. The scheme could also aid evaluation of new control measures, e.g. vaccination or management changes, not only at farm-level but at a national level. For example within the context of pig farms considered in the current study, BPHS reports could be used more efficiently by producers, veterinarians and BPEX to assess the effectiveness of vaccination against PCV2 at reducing disease in slaughter pigs.

The contribution BPHS data can make to the British pig industry depends on the coverage of farms, the more producers enrolled in the scheme the more representative the data. Only specialised abattoirs offer the scheme, participation is voluntary and producers have to pay membership fees [15]. From July, 2011 all assured English pig producers who register for membership of the National Pig Health Improvement Project (http://www.pighealth.org.uk/health/home.eb) will receive free membership to the BPHS scheme and the number of abattoirs offering the scheme has increased. Although the sample is still biased towards large producers these incentives should increase participation to some extent.

## Conclusions

This study suggests that the lesion scores reported by BPHS may reflect the presence of respiratory pathogens on the farms and these lesions may be indicative of reduced productivity. However, not all respiratory pathogens were included in the study and pigs tested for respiratory pathogens were not the same pigs that were examined by BPHS. Further studies in slaughterhouses, where laboratory testing is performed on pigs that are being examined by BPHS and which test for more respiratory pathogens, are needed to evaluate the scheme fully.

## Authors' contributions

PAL, MV and BW designed the cross-sectional study and PAL and MV carried out the farm visits and data collection. HRH, BW and DP developed the approach for the present study. PAL assisted with statistical analysis and HRH performed statistical analysis and drafted the manuscript. PAL, BW and DP critically revised the manuscript and all authors read and approved the final manuscript.
